# Establishing medical plausibility in the context of orphan medicines designation in the European Union

**DOI:** 10.1186/s13023-014-0175-8

**Published:** 2014-12-05

**Authors:** Stelios Tsigkos, Segundo Mariz, Jordi Llinares, Laura Fregonese, Stiina Aarum, Naumann-Winter Frauke, Kerstin Westermark, Bruno Sepodes

**Affiliations:** Orphan Medicines Office, European Medicines Agency, 7 Westferry Circus, Canary Wharf, E144HB London, UK; Bundesinstitut für Arzneimittel und Medizinprodukte, Kurt-Georg-Kiesinger-Allee 3, 53175 Bonn, Germany; Läkemedelsverket, Dag Hammarskjölds vägen 42, 75103 Uppsala, Sweden; Research Institute for Medicines (iMED.ULisboa), Faculty of Pharmacy, University of Lisbon, 1649-003 Lisboa, Portugal

## Abstract

In the European Union, sponsors have the responsibility to demonstrate the “intention to diagnose, prevent or treat” a serious and rare condition before the Committee of Orphan Medicinal Products (COMP), for a medicinal product to meet the criteria for Orphan Designation. This requirement is commonly referred to as “medical plausibility” and the justification of this intention is assessed on the merits of each application by the COMP, which deliberates over the scientific evaluation of the evidence submitted. The scientific assessment of the applications for orphan designation by the Committee is based on the review of non-clinical (such as *in vitro* and *in vivo*) and/or clinical data submitted by the sponsor. Several challenges regarding the evidence provided emerge when the sponsor is applying for a designation at an early stage of development. Herein we discuss specific examples from the experience of the COMP, in order to elaborate on the type and level of evidence generally considered necessary for the purpose of justification of the intention to treat an orphan condition. Importantly, it is pointed out that bridging of data from other products, irrespectively of how comparable they may be, or from settings not directly associated with the condition as applied for designation, is by and large not a successful exercise and may only be exceptionally considered. It is further exemplified that, as reflected in the updated *‘Guideline on the format and context of the applications for designation’* and the guidance document ‘*Recommendation on elements required to support the medical plausibility and the assumption of significant benefit for an orphan designation’* available on the EMA website, the sponsor should provide data with the specific product as applied for in specific models of the condition or in patients affected by the same condition subject of each application.

## Background

In order to incentivise the development of medicinal products for rare conditions that would not otherwise attract attention by the pharmaceutical industry, a European framework for “orphan medicinal products” has been put in place for more than a decade now. The Committee of the Orphan Medicinal Products (COMP) of the European Medicines Agency (EMA) evaluates the applications for Orphan Medicinal Product designation in the EU. The criteria for orphan designation in the EU are described in Article 3 of Regulation (EC) No. 141/2000 [1]. As per the first paragraph of Article 3(1) of the abovementioned regulation, it is provisioned that a medicine shall be designated as an orphan medicinal product if its sponsor can establish: *“that it is intended for the diagnosis, prevention or treatment of a life-threatening or chronically debilitating condition affecting not more than five in 10 thousand persons in the Community when the application is made (…)*”.

In the EU orphan regulatory practice, this intention to diagnose, prevent or treat is also referred to as “medical plausibility”, and as such cited in the respective guideline, which expects data to be presented by the sponsors to confirm the feasibility of the proposal [2,3]. In this manuscript the terms “intention to treat”, as appearing in the orphan regulatory jargon and “medical plausibility” are used interchangeably. The reader is advised not to confuse this “intention to diagnose, prevent or treat” with other homophone concepts such as populations of clinical trials.

To justify medical plausibility before the COMP, the sponsor has to present data from studies with the product which support the promise of a relevant effect in the specific condition proposed for designation. Providing data of this nature is not always straightforward, given that the medicinal product may be at an early stage of development and the data comes from non-clinical models which are difficult to interpret [4]. Indeed, Article 5 specifies *“In order to obtain the designation of a medicinal product as an orphan medicinal product, the sponsor shall submit an application to the Agency at any stage of the development of the medicinal product before the application for marketing authorisation is made”*.

A “tug-of war” may therefore be conceptualised between the need for data to support the criteria for either diagnosis, prevention or treatment of the condition on the one hand, and the provisions allowing sponsors to seek designations at very early developmental stages on the other, thereby relying on results that need extrapolation and acceptance of pharmacological assumptions in the assessment process by the COMP.

Since 2000 [5], the issue of medical plausibility has been extensively deliberated and has evolved into a firm practice of the data requirements associated with establishing medical plausibility. This is reflected in the fourth revision of the *Guideline on the Format and Content of Application ENTR6283/00 Rev 04 2014* [2]. Under the medical plausibility section on page 6, it is stated, *“In order to support the rationale for the development of the product in the proposed condition preliminary preclinical or some clinical data are generally required. It is important to include, as far as possible, a discussion of the results of pre-clinical studies with the specific product, as applied for in the specific condition, and/or a discussion on preliminary clinical data in patients affected by the condition. Where available, reports of studies from the sponsor and supporting the use of the product in the applied condition should be included in the orphan designation application. The aim, methodology, results of all relevant studies, etc., should be submitted at the time of the application”.*

Since September 2012 the EMA, following its policy on transparency, started to publish the COMP meeting minutes. This manuscript discusses some orphan designation cases which have been discussed in the published minutes found on the Agency’s website, with the intention to provide hands-on examples that may help future sponsors and interested parties to understand the data requirements for the justification of medical plausibility. This is without pre-empting the COMP’s remit to discuss each application on the basis of its own scientific merits.

## Methods

### Case studies from the COMP procedures of the year 2013

The published minutes of the year 2013 [5] were reviewed for examples of data submitted for the evaluation and outcome of medical plausibility for the purpose of Orphan Medicinal Product Designation. This year was chosen for most current examples that would reflect the evolved standards in the consideration of medical plausibility, as also reflected in the recently updated guideline [2].

The starting point for selecting examples was the consolidated experience on the common issues raised during the procedures of the COMP, with regards to justification of medical plausibility. These issues were used as the basis to form the categories for the purpose of this paper, as outlined below. The categories were then populated by case studies from the published minutes, and several excerpts from these minutes are also included in the discussion [5].

It has to be noted that the selected examples focus exclusively on the issues associated with data submitted for the purpose of establishing the medical plausibility issues and not any other orphan criteria. The sponsors, in addition to the medical plausibility, have to justify that all criteria of the orphan Regulation are met; nevertheless, those rest outside of the scope of this paper. It is finally stressed that the cited examples were selected merely on the basis of clarity, simplicity and educational usefulness for demonstrating the points of this reflection and not on the base of any other merit and could have been equally replaced by other examples.

## Results

### Examples of successful justification of medical plausibility

#### Justification based on preclinical data

Preclinical data have been used in establishing medical plausibility within the context of an orphan medicinal product designation. The scientific rationale for use of the product in the applied condition is to be elucidated as far as possible. In this type of submissions the most decisive points within the framework of the evaluation process are:

- the relevance of models used, and
- the relevance of endpoints studied.

The models used should replicate the features of the medical condition as closely as possible, in order to allow for extrapolations to be made and to draw conclusions for the condition as applied for designation. It follows that early preclinical studies, such as *in vitro* studies alone, would be more difficult to interpret than higher level studies such as those performed in validated animal models of disease. As per the COMP recommendation paper on elements required to support the medical plausibility and the assumption of significant benefit (EMA/COMP/2009) *‘at least relevant in vitro and in vivo data in appropriate preclinical models should be submitted’*, stressing the need for adequate studies to have been performed in animal models [3]. Only exceptionally may in vitro data be considered as a sole basis, and as per the same recommendation paper “if in vitro studies only is available…the relevance of the findings should be discussed in the context of the proposed condition”. For example, in the case of a well-known condition and a well-known class of products, in vitro data in cell lines might be considered supportive.

Moreover, the endpoints chosen in the preclinical studies should also be relevant to the clinical target sought, thereby aiding evaluators to make a meaningful assessment of relevant improvements secondary to the pharmacological intervention.

The relevance of models used and the relevance of endpoints studied, should therefore be viewed as necessary prerequisites for any application in support for medical plausibility. It is important to point the difference between applications based on scientific hypothesis where a products’ mode of action in the target condition is presented with support from a generic scientific rationale on the one hand, and applications based on data with the proposed medicine in a relevant model of the condition and endpoints which are indicative of effect. This is reflected in the following examples [5].

#### Case 1. High altitude pulmonary oedema

High Altitude Pulmonary Oedema is a life-threatening form of non-cardiogenic pulmonary oedema that develops in susceptible people who ascend quickly from low to high altitude, typically above 2.500 meters [6]. Its pathophysiology remains unclear, but low partial pressure of oxygen at high altitude causes excessive hypoxic vasoconstriction, inadequate ventilatory response to hypoxia, and leak or stress failure of the pulmonary capillaries. It remains the major cause of death related to high-altitude exposure, with a high mortality rate in the absence of adequate emergency treatment [6,7]. High altitude pulmonary oedema is a rare condition in the European Union with an estimated incidence of less 0.03 in 10,000 people [7].

In an application for the treatment of high altitude pulmonary oedema, the Committee was presented with studies of the candidate product in preclinical models, which recapitulated specific features of the condition. Relevant in vivo non-clinical studies included a mouse alveolar flooding model and a rat high altitude pulmonary oedema model. Available data from these preclinical studies showed increased alveolar clearance, as well as progressive recovery of relevant functional parameters including dynamic lung compliance and airway resistance. Because of the relevance of both models used, which recapitulated aspects of the pathophysiology of the condition, and the outcomes studied, the medical plausibility was considered justified.

#### Case 2. Niemann pick

Niemann-Pick is a group of lysosomal lipid storage disorders, with visceral and neurological manifestations. A recent peer review [8] divides this condition into two entities: *i*) acid sphingomyelinase-deficient disease resulting from mutations in the SMPD1 gene (encompassing type A, B and intermediate forms), and *ii*) Niemann-Pick disease type C (including also type D), resulting from mutations in either the NPC1 or the NPC2 gene. Niemann-Pick type C has been considered by the COMP to be affecting approximately 0.1 in 10,000 people in the EU [5].

Two applications have received a positive opinion in 2013 for the treatment of Niemann-Pick type C (NPC). In one application, the sponsor discussed relevant studies in preclinical settings including the NPC1−/− mouse model [9]. The NPC1−/− model is a well characterised mouse model for NPC: the pathology comprises accumulation of cholesterol and glycosphingolipids and the brain, liver and spleen are affected, while the affected mice die at approximately 11 weeks. In this model, treatment resulted in lower levels of glycosphingolipids in the brain and liver compared to the untreated controls, as well as inhibition of the accumulation of unesterified cholesterol in the liver and kidney. In addition to these biochemical markers, improved motor performance as per rearing activity scoring and gait analysis was reported. For the second application, medical plausibility was considered acceptable based on data generated in the same NPC1−/− mouse model, as well as in a feline model of the condition, in cats with a spontaneously occurring missense mutation in NPC1 gene [10]. The feline model of NPC has been characterized and is phenotypically, morphologically, and biochemically similar to human NPC1 [11]. In that model treatment showed even improvements in neurological symptoms and survival.

In both cases, the relevance of the models used and the endpoints for the actual clinical entity under review, allowed for the medical plausibility to be established.

#### Case 3. Duchenne Muscular Dystrophy (DMD)

Duchenne muscular dystrophy (DMD) is an X-linked inherited disorder characterized by progressive muscle weakness, wasting and degeneration. Although the gene affected in DMD was identified over 25 years ago, there is still no effective treatment [12] and the condition has been designated in the European Union as a chronically and seriously debilitating condition affecting approximately 0.5 in 10,000 people [5]. Most children affected by the condition will need a wheel chair before 12 years of age, while respiratory muscle deterioration results in reduced forced vital capacity of the lungs, requiring ventilation support. Death occurs at median age of 25 years, usually due to respiratory or cardiac failure.

Applications for the treatment of DMD based on relevant preclinical models, such as the dystrophin-null mdx dystrophic mouse model, are common [13]. In fact, all three applications that received a positive opinion in 2013 included data from studies using the mdx mouse model. With regards to the considered endpoints, in one successful designation case, the sponsor submitted data showing improved grip strength and inflammation in the mdx mouse model. In another, improvements in lean muscle mass and grip strength was presented to the Committee. In a third successful case in 2013, the sponsor presented studies in the mdx model, showing improvements in muscle strength, histology and inflammation; moreover the sponsor also included data in a double knockout murine model (dKO) of utrophin and dystrophin, showing improved muscle strength and histology, while treatment also improved lifespan of animals.

Again the COMP, having considered both the validity of the models and the clinical relevance of the endpoints studied, considered that the intention to treat DMD had been justified by the respective sponsors.

### Justification based on applications including preliminary clinical data

While orphan designation based on solely preclinical data is possible at early stages of development, the level of assumption may be reduced when successful preliminary clinical studies are included in the application. The degree of assumption is still considerable, taking into account that properly conducted clinical studies may not be available or feasible at that point in time and that confirmation of efficacy is not required at this stage. In applications defended on the grounds of preliminary clinical data, the discussion revolves around the population studied in terms of representativity for the applied disease, other treatments that may be received by the patients, the design and in particular the availability of controls, the endpoints assessed and the relevance of the results observed in the treated patients. Before considering such data, the possibility for placebo effects, spontaneous recovery of patients or critical bias is always scrutinized. The rationale to use the product in the applied condition with regards to the mechanism of action should also be elucidated as far as possible. The examples discussed below illustrate that if a sponsor can show clinically relevant improvements in even a limited number of treated patients, the intention to treat may be considered justified.

#### Case 4. Adenonsine-deaminase deficient severe combined immunodeficiency (ADA-SCID)

Severe combined immunodeficiencies (SCID) are a group of diseases caused by monogenic disorders that impair T-cell development and can be associated with faults in the development of other hematopoietic lineages [14]. At least ten different molecular causes have been described; deficiency of adenosine deaminase (ADA), an enzyme of the purine salvage pathway, accounts for up to 20% of the total number of cases [14].

It is difficult to distinguish between the different forms of SCID on the basis of clinical presentation alone. Children with SCID lack immune protection from infections and are prone to repeated and persistent bacterial, viral and fungal infections that can be very serious or life-threatening. In its most severe form, ADA-SCID is fatal within the first year of life. In the EU ADA-SCID has been estimated to affect less than 0.1 in 10,000 people [5].

In an example of an application for the treatment of ADA-SCID with a gene therapy product, the sponsor presented both preclinical and clinical studies. The in vitro studies showed ADA expression in CD34+ cells from patients as a result of treatment, while in vivo the product was shown to engraft in valid pre-clinical models of the disease which used the ADA−/− mouse model and the NSG/NOD/SCID/gamma c−/− mice, resulting in expression of ADA by immune cells. Moreover, the compassionate use of the product in patients with severe ADA-SCID resulted in effective gene transduction and in T cell recovery and metabolic correction to the date of application for designation, at 6 months of observation. Based on these preclinical data in relevant models of the condition and preliminary clinical data in affected patients showing relevant outcomes, the medical plausibility was considered justified for the purpose of orphan designation. The well-defined mode-of-action allowed the consideration even of case reports for the justification of medical plausibility.

#### Case 5. Eosinophilic oesophagitis

Eosinophilic esophagitis is a chronic immune-mediated condition where infiltration of eosinophils into the esophageal mucosa leads to esophageal dysfunction [15]. It is a rare condition previously designated by the COMP with a prevalence of less than 5 in 10,000 people in the EU [5].

In an application for eosinophilic esophagitis, the intention to treat the condition with the medicinal product was considered justified based on clinical trials showing histologic response and reduction of symptoms in adult and paediatric patients treated with the product. Several trials were discussed from the available literature, which were supplemented by sponsor-generated data from a phase I and an on-going phase II study showing histological remission in affected patients. In a second application, the medical plausibility was considered justified based on preclinical and preliminary clinical data from a small randomised controlled trial in adult patients with the target condition, showing reduction of eosinophil numbers in the oesophageal mucosa. This effect was considered clinically relevant as the condition is characterised by eosinophilic inflammation.

#### Case 6. Epidermolysis bullosa

Epidermolysis bullosa (EB) is a heterogeneous group of inherited skin diseases characterized by increased skin fragility and variable degrees of extra-cutaneous presentations. The four major types are simplex EB, dystrophic EB, junctional EB, and Kindler syndrome [16]. The severity of the condition ranges from localized blisters and erosions to debilitating deformities and severe gastro-intestinal involvement.

The COMP has considered that EB is chronically debilitating and life-threatening, and affecting less than 0.8 in 10,000 persons in the European Union [5].

In an application for the treatment of EB, the sponsor defended the medical plausibility on preliminary clinical observations from compassionate use programs and a small study of eight patients. In the latter setting, the sponsor reported favourable outcomes in the wound healing of EB patients. The sponsor further supported its position by preclinical literature data on the general effects of the product in wound healing.

As per the published minutes, the Committee discussed whether the results from other wounds can be extrapolated to the specific condition subject of the application, and whether the clinical data presented could be considered without a clear pharmacological target for the product. The Committee was of the view that the extrapolation using not exactly the same product but another formulation with different concentrations of the active substance than the one proposed was of limited value. In addition, with regard to the clinical studies, the course of the condition is reported to “cycle”, i.e. there is a loss of efficacy of skin care products after longer use. The initial response of the patients in the uncontrolled setting may therefore have been observed simply be due to the change of product.

Despite these reservations, it was acknowledged that the sponsor was developing a specific product and there were clinical observations that support the activity of the product in the context of treatment of EB. This was considered sufficient at that stage to support the medical plausibility in the context of an orphan designation.

#### Case 7. Glioma

Glioma is a CNS neoplasm arising from glial cells and includes several subtypes depending on the type of cells that give rise to the pathology. Malignant glioma comprises glioblastoma (WHO grade IV), anaplastic astrocytoma (grade III), mixed anaplastic oligoastrocytoma (grade III) and anaplastic oligodendroglioma (grade III), with glioblastoma carrying the worst prognosis [17]. Glioma has been the subject of a plethora of applications for orphan designations and the COMP has previously considered that the condition is chronically debilitating, in particular due to compression and invasion of the surrounding brain structures leading to neurological deficits, and life-threatening with poor overall survival, which in particular for glioblastoma multiforme patients is less than 5% at 5 years post diagnosis. The condition was estimated to be affecting less than 3 in 10,000 persons in the European Union [5].

In an orphan designation procedure discussed for the treatment of glioma in 2013, the sponsor defended the medical plausibility on both preclinical and preliminary clinical data. In the preclinical setting, the sponsor studied the effects of the product in murine models that have been xeno-transplanted with glioma cell lines. In these models, tumour growth was inhibited by treatment with the compound. In addition, as for the preliminary clinical studies, the sponsor discussed a Phase 1 dose-escalation study in patients with recurrent malignant glioma and showed responses with regards to tumour size. The COMP therefore accepted the medical plausibility based on both preclinical and clinical data.

### Examples of unsuccessful efforts to justify medical plausibility

As reflected in the updated guideline on the format and content of applications for orphan medicinal product designation [2], the data required for the demonstration of medical plausibility should be “as far as possible specific for the proposed product and the proposed condition, either in relevant models or in patients affected by the specific condition”.

In the absence of such data the applications remain by large unsuccessful, because bridging to data from other settings increases the assumption level and weakens medical plausibility. Only exceptionally, if objective limitations can be documented, as in the case where cross-species specificities necessitate the use of a non-human surrogate product to be used in models of the condition, the committee has considered such extrapolations.

The examples discussed below exemplify the principle that in the absence of data generated with the product in the condition as applied for designation, orphan status is considered difficult to be granted. It is to be noted that the publicly available information on these procedures is more limited [5] compared to the examples discussed above, because of confidentiality issues.

### Bridging to other products

#### Case 8. Alagille syndrome

Alagille syndrome is an autosomal dominant disorder associated with dysfunction of the liver, heart, spine, and eyes, which is also characteristic by a distinctive facial appearance in many patients. It is associated with the defect in component of the Notch signaling pathway, with mutations in JAG1 (type 1) or NOTCH2 (type 2) [18]. The main clinical features are chronic cholestasis due to paucity of intrahepatic bile ducts, congenital heart disease primarily affecting the pulmonary outflow tract and vasculature, “butterfly” vertebrae or other abnormal vertebral segmentation, characteristic faces with a broad forehead, and ophthalmic disorders such as posterior embryotoxon, anterior segment abnormalities, and retinopathy. Additional features are intracranial bleeding and dysplastic kidneys. The diagnosis is essentially clinical, and therapy is focused on the consequences of liver disease, as well as the surgical and medical treatment of congenital heart defects [19].

In the context of orphan designation, the condition has been previously considered by the Committee to be chronically debilitating due to hepatic and cardiac dysfunction. Portal hypertension develops in up to one third of patients and life expectancy is in most cases is around 20 years and death is associated with liver failure, cardiac problems and blood vessel abnormalities. The condition has been estimated to be affecting not more than 0.3 in 10,000 persons in the European Union [5].

As reflected in the published minutes, in an application of a product for the treatment of Alagille syndrome, the Committee considered the available preclinical in vivo data to support the intention to treat, as presented by the application, pertained mainly to another surrogate product (even though with the same mechanism of action) which could not be directly extrapolated to draw conclusions for the specific condition under evaluation. Therefore the data were not considered appropriate to justify the medical plausibility.

#### Case 9. Schnitzler’s syndrome

Schnitzler's syndrome is a disorder characterized by recurrent fever and urticarial rash, muscle bone or joint pain, lymphadenopathy and monoclonal gammopathy. It is considered as a paradigm of an auto-inflammatory syndrome, of which fewer than 250 patients known, even though the number is probably higher [20].

In the case of a medicinal product proposed for Orphan Designation for the treatment of Schnitzler’s syndrome, the applicant did not present data with their own product in relevant settings but attempted bridging to data obtained with other products having the same mechanism of action. Having identified this limitation, the COMP invited the sponsor to present data of their own investigations with the product, in a pre-clinical model or in a preliminary clinical setting in the condition. The sponsor eventually withdrew the application.

### Bridging to non-relevant models, or other conditions

#### Case 10. Recurrent HCV infection in liver transplant recipients

In recurrence of hepatitis C virus (HCV) infection following liver transplantation, the progression in the allograft is more severe and develops faster compared to other HCV infection settings. In the context of acceptability as an orphan condition by the COMP, this difference has been previously considered as salient for discerning recurrence in transplant recipients from other HCV infections.

About one third of HCV-infected recipients have developed allograft cirrhosis due to HCV recurrence by the 5th-7th year post-transplantation [21]. In several previous cases, the COMP has considered that recurrence of HCV infection in liver transplant recipient was affecting less than 0.1 in 10,000 persons in the European Union, and confirmed the chronically debilitating and life-threatening nature of the condition, in particular due to hepatic complications including cirrhosis and hepatocellular carcinoma [5].

In a COMP procedure discussed in 2013, a sponsor initially proposed a product for orphan designation for prevention of recurrent HCV infection in liver transplant recipients. The application was based on data stemming from studies in the context of treatment of chronic HCV infection, and not from studies in the condition as applied for designation, which was treatment of recurrent HCV infection in liver transplant recipients Having examined the application, the Committee considered that recurrence of HCV in liver transplant recipients is a distinct population compared to chronic HCV infection in non-transplant recipients, and because of this, the biochemical endpoints discussed did not allow for an extrapolation to the sought indication as applied for designation. Thus the medical plausibility was not considered acceptable in the absence of data in relevant settings.

### Inadequate results in relevant settings

Interestingly, even if appropriate settings and endpoints are used, the results obtained in the studies of an orphan designation dossier may still be difficult to interpret. The actual results obtained remain the major decisive factor in the evaluation of medical plausibility. Some points are of particular importance: first, the extent of effects caused by the product may be limited, putting into question the clinical relevance of the proposed intervention. Second, the uncontrolled nature of some studies, as not infrequently presented to the COMP in early development stages, prevents the attribution of properties to pharmacological products. This is particularly difficult in the context of a weakly defined mechanism of action of the product or a potentially fluid course and phenotypic variability of the clinical condition. Third, administration of the product in combination with other products further complicates the generated observations. This is exemplified in the case discussed below.

#### Case 11. Fragile-X syndrome

Fragile X syndrome is considered to be the most common cause of intellectual disability and autistic spectrum disorders [22]. The condition is caused by transcriptional silencing of the FMR1 gene. The product of this gene, FMRP, is an RNA-binding protein, which is theorised to have a critical role in targeting neurospecific mRNAs to brain synapses and inhibiting protein synthesis in response to synaptic stimulation signals [23]. Largely based on work in the fmr1 knockout mouse model, the condition has emerged as a disorder of synaptic plasticity associated with abnormalities of long-term depression and long-term potentiation and immature dendritic spine architecture [24].

The COMP has previously designated orphan products for the treatment of fragile X-syndrome, with the condition estimated to be affecting approximately 2 in 10,000 persons in the European Union, and being chronically debilitating in particular due to neuro-behavioural and neurodevelopmental symptoms including cognitive impairment, anxiety, irritability, social withdrawal, inattention and hyperactivity, as well as epileptic seizures [25].

In a recent case of an application for the treatment of Fragile-X syndrome which discussed in 2013, the sponsor presented data in a preclinical model of the condition using FMR1 deficient mice, as well as preliminary clinical data in patients affected by the condition. With regards to the clinical observations, the sponsor argued improvements after treatment with the proposed product based on symptom rating scales. The Committee questioned the relevance of the endpoints used, and considered that given the open nature of the studies and the concomitant treatment with other psychotropic medications, it would be difficult to draw conclusions attributable to the proposed product. Moreover, a lack of clarity regarding the mode of action of the proposed product was identified. It was therefore considered that the data included in the application were not sufficient to justify the intention to treat the condition with the applied product.

## Discussion

The COMP assesses all applications submitted for orphan desigantion on a case by case basis; it has to be noted that the level, quality and quantity of data presented with each application varies significantly. This depends on the particularities of the condition and product, as well as the stage of development which may be at any point prior to submitting a Marketing Authorisation Application. In practice, the scientific support put forward may range from a description of the assumed mode of action of a product in the target condition to detailed analysis of endpoints from interventional studies in affected patients. However, a generic scientific rationale, should generally be further substantiated with specific data from the condition as applied for designation, either in relevant models (preclinical) or from patients with the disease, unless the absence of such data can be adequately justified. This expectation to provide data with the product in the condition is clearly reflected in the updated relevant guideline [2].

Accordingly, when reviewing the available data to justify medical plausibility, three main points are of particular importance; the availability of data with the specific product as applied for designation, the relevance of the non-clinical in vivo models or target patient population used for the studies, and lastly the clinical relevance of endpoints and results obtained. All of these aspects contribute towards minimising the level of assumption required, relative to the stage of development. Thus, a balanced evaluation based on the level of evidence can be made to allow for justification of the intention to treat, diagnose or prevent a condition at the time of designation. It could therefore be conceptualised that a level of “maximum assumption level” relative to the stage of development exists, above which the medical plausibility cannot be accepted. This assumption level may be reduced, if data with the product in the condition show meaningful improvements in relevant endpoints.

The first prerequisite is to refer to a specific medicinal product, which the sponsor is developing for a specific orphan indication. Notwithstanding that a sponsor may apply at any stage of development [1], it is usually the case that the development of the specific product has commenced and is underway. Hence, the sponsor should have in general performed some non-clinical and/or preliminary clinical studies with the proposed product as applied for designation.

If no data are available with the proposed product under review the level of assumption is so high, that renders the application by large “hypothetical”. As noted above, the pharmacological target may present an opportunity based on strategy and scientific rationale in general, but without any data with the specific product, the establishment of the orphan criteria is exceptionally difficult to consider. In line with this notion, the Committee has previously requested in several procedures confirmation that the product actually exists, and questioned whether it will be developed in the sought indication based on the absence of data in preclinical or clinical settings.

The second prerequisite would be the use of the specific product in a relevant non-clinical model and/or preliminary clinical data in a target patient population with the condition. The experience of the COMP with regards to the potential and limitations of animal models in the facilitation of drug development in rare diseases has been previously discussed in the literature, where models for metabolic, neuromuscular and ophthalmological orphan-designated conditions have been presented [13]. In the examples discussed above, it is also pointed out that in the absence of such data the applications remain by large unsuccessful, because bridging to data from other settings increases the assumption level and weakens medical plausibility. However exceptionally, if objective limitations can be documented the committee has considered such extrapolations.

Thirdly, clinically relevant endpoints are to be studied, and the results obtained should be permissive of a clinically relevant intervention. In early stages of development, the acceptability of endpoints may also be considered in the context of a valid pathophysiologic pathway known to be implicated in the disease. Moreover, even if appropriate models or clinical settings are used, the results should still be convincing to support the sought indication. The extent of effects should be clear and the observations have to be confidently attributed to the product.

## Conclusions

Based on the information from the COMP practice, as it is reflected in the published minutes of its plenary meetings, and in the context of the provisions of the orphan framework and the relevant available guidance, there are three points that in general need to be met in order to justify the medical plausibility:

- data with the specific product as applied for designation are required; the proposed product exists and a stage of development can be identified;
- results from studies with the product are required in either specific models of the condition, or in patients affected by the condition as applied for designation;
- the endpoints studied have to be clinically relevant and the results adequate to allow for scientific claims on improvement upon administration of the product under review.

In conclusion, notwithstanding that the sponsors may apply for orphan medicinal product designation in the EU at any stage of development, the applications are expected to include adequate data to support the proposal: the justification of medical plausibility can be straightforward, provided that relevant results exist in the settings of the specific condition and with the specific product as applied for designation. Bridging of data from other products, or other conditions than the one under review is by and large not successful.
