# Erratum to: Establishing medical plausibility in the context of orphan medicines designation in the European Union

**DOI:** 10.1186/s13023-015-0309-7

**Published:** 2015-10-06

**Authors:** Stelios Tsigkos, Segundo Mariz, Jordi Llinares, Laura Fregonese, Stiina Aarum, Frauke Naumann-Winter, Kerstin Westermark, Bruno Sepodes

**Affiliations:** Orphan Medicines Office, European Medicines Agency, 7 Westferry Circus, Canary Wharf, London, E144HB UK; Bundesinstitut für Arzneimittel und Medizinprodukte, Kurt-Georg-Kiesinger-Allee 3, Bonn, 53175 Germany; Läkemedelsverket, Dag Hammarskjölds vägen 42, Uppsala, 75103 Sweden; Research Institute for Medicines (iMED.ULisboa), Faculty of Pharmacy, University of Lisbon, Lisboa, 1649-003 Portugal

## Erratum

After publication of [1] it came to the authors’ attention that Frauke Naumann-Winter’s name was displayed incorrectly. The correct spelling of their name is included in this erratum.
